# Formamidinium lead iodide perovskite photovoltaics with MoS_2_ quantum dots

**DOI:** 10.1038/s41598-024-72037-3

**Published:** 2024-09-16

**Authors:** Ankur Uttam Kambley, Bruno Alessi, Calum McDonald, Pagona Papakonstantinou, Vladimir Svrcek, Davide Mariotti

**Affiliations:** 1 School of Engineering, Ulster University, York Street, Belfast, BT15 1ED UK; 2 Renewable Energy Research Center, National Institute of Advanced Industrial Science and Technology (AIST), Tsukuba, Ibaraki 305-8568 Japan; 3 Department of Design, Manufacturing & Engineering Management, University of Strathclyde, Glasgow, UK

**Keywords:** Materials for devices, Nanoscale materials

## Abstract

We present the formation of a composite film made out of formamidinium lead iodide (FAPI) and molybdenum disulphide quantum dots (MoS_2_ QDs) and propose a corresponding photovoltaic device architecture based on a ‘*type-I*’ alignment of the two materials’ electronic energy levels. The introduction of the MoS_2_ QDs has not compromised the overall crystallinity of the FAPI film and the composite absorber has shown improved stability. We report on the benefits of this composite film and energy band arrangement as the photogenerated carriers in MoS_2_ QDs, both positive and negative, are injected into the FAPI host matrix, resulting in an increased current density of 24.19 mA cm^−2^ compared to a current density of 19.83 mA cm^−2^ for the control device with FAPI only. The corresponding photoconversion efficiency increases from 12.6 to 15.0%. We also show that inclusion of MoS_2_ QDs in FAPI films resulted in a notable improvement in the fill factor and open-circuit voltage of the solar cells. Most importantly, MoS_2_ QDs enhanced the film stability by reducing defect formation and acting as passivating agents that minimize recombination losses and improve charge carrier transport. Our results suggest that a composite film in a *type-I* device architecture can introduce benefits for both future developments in perovskite solar cells and effectively tackling the longstanding challenges of carrier transport in QDs solar cells.

## Introduction

The development of next generation solar cells has witnessed tremendous progress in the past 15 years with the introduction of organometal halide perovskites (OHP) as contenders to silicon-based absorbers. Initial efforts in perovskite solar cells (PSCs) yielded certified efficiencies below 15% in 2013, well below silicon-based technologies^1^. Fortunately, despite the much lower efficiencies, scientific progress was supported by the research community, with PSCs now offering a viable alternative to current commercial photovoltaic technologies.

Today, PSCs are close to match the performance of well-established silicon based cells or reach even higher efficiencies in tandem with silicon^1–4^. In addition, the low cost of solution-processing of OHPs makes it an attractive choice for manufacturing. Among PSCs, devices with formamidinium lead iodide (FAPI, CH_3_(NH_2_)2PbI_3_) have shown a very competitive performance^5^. Pb-based OHPs are attractive for photoconversion applications due to their high absorption coefficients^6^, non-radiative recombinations^7^, long carrier diffusion lengths^8^, bandgap tunability and low exciton binding energy^9^. Despite the advantages, Pb-based PSCs have poor shelf life compared to already established silicon-based solar cells because of their sensitivity to moisture and oxygen, which induces degradation of the perovskite film into lead iodide (PbI_2_)^10,11^. The replacement of Pb is always at the expenses of device performance and, the possibility of boosting photocurrent, while using lead-free perovskites, is an attractive and active research topic.

Substantial progress has been made also in other photovoltaic technologies, and for instance researchers have made impressive steps forward in both organic solar cells as well as in quantum dots (QDs) solar cells^1,12,13^. Since 2013, organic solar cells have improved their efficiencies from below 12% to above 19% while QD solar cells have reached also efficiencies above 19% with an impressive jump from below 8%^1^. Furthermore, QD solar cells, which have shown improvements in par with PSCs, do offer the additional potential of overcoming the Shockley-Queasier limit through carrier multiplication^14,15^. However, despite very impressive efforts^16^, one of the main obstacles of QD solar cells remains the ‘complicated’ carrier transport. In current QD device architectures, carrier transport is necessarily limited by boundary interfaces of the QDs as devices rely on the formation of QDs percolating networks and type-II band alignment (e.g. single-junction, bulk heterojunction)^14^.

In order to overcome the limitations of current QD devices, we have recently introduced the possibility of exploiting ’type-I’ alignment of energy levels^11^, therefore suggesting a different approach to QD solar cell architectures that can facilitate the exploitation of carrier multiplication. At the same time, the new device architecture with QDs, represent a new avenue that could also introduce important opportunities in perovskite technologies.

In the proposed ‘type-I device architecture’, the wider bandgap QDs are localised and distributed inside a narrower bandgap bulk semiconductor host material, which together form a composite absorber layer of the solar cells without the requirement for QDs to form percolating networks. The QDs and the semiconductor host material are expected to form an ‘atypical’ band alignment resulting in a type-I heterojunction. We previously demonstrated the use of silicon nanocrystals as the wider bandgap QDs and methylammonium lead iodide (MAPI) OHP films as narrower bandgap material, which resulted in a current density (J_SC_) of 16 mA cm^−2^ compared to a J_SC_ of 10 mA cm^−2^ for a MAPI-only absorber film^11^. Another interesting property of type-I architecture is the possibility of electronic and optical coupling internally in the absorber layer which could lead to carrier multiplication^17–20^. The electronic-coupling occurs when both carriers are generated from both the QDs and the host material via direct photoexcitation and the carriers are extracted from the QDs to the narrower bandgap host material due to type-I alignment. Alternatively optical-coupling occurs when the photoexcited carriers in the QDs go through radiative recombination which releases energy in the form of photons and subsequently these photons cause photoexcitation of carriers in the surrounding narrow-bandgap material. In both coupling cases, the carriers extracted from the absorber layer are a combination of carriers generated from both the QDs and the narrow bandgap material. Therefore, type-I architecture provides a novel approach for solar cell performance enhancement drastically reducing the restriction and limitation of current QD solar cells.

Molybdenum disulphide (MoS_2_) is an attractive material in this context as it exhibits an exceptionally high absorption coefficient and fast carrier dynamics leading to lower recombination rates^21,22^. Bulk MoS_2_ is reported to have a bandgap of 1.2 eV while quantum confined MoS_2_ nanostructures, with a larger bandgap, have also been explored for a range of applications^23–28^. The vertical alignment of MoS_2_ layers in MoS_2_ QDs is also reported to assist in perovskite crystallization resulting in enhanced crystallinity and passivation of interfacial defects leading to enhanced performance of the solar cell^29^. Inclusion of MoS_2_ nanosheets in perovskite film has also shown to promote oriented crystal growth and high crystal-phase purity reducing the lattice stress of the perovskite film^30^, resulting in higher photovoltaic performance and overall shelf lifetime. A commonly used hole transport layer in PSCs is lithium (Li) doped Spiro-OMeTAD films. MoS_2_ nanocrystals have also been reported to be an absorbent of Li^+^ ions, thereby suppressing migration of Li^+^ ions from the hole transport layer to the perovskite layer resulting in reduced current hysteresis and increased open-circuit voltage (V_OC_)^31,32^. Therefore, MoS_2_ nanostructures have been proved to be beneficial in a wide range of aspects for PSCs, including film crystallisation, interface passivation and hysteresis suppression. These aspects make MoS_2_ nanostructure a prime candidate that could prove highly beneficial in a type-I device architecture within a FAPI layer.

In this paper, we demonstrate the feasibility and successful fabrication of stable FAPI/MoS_2_ QDs composite films and provide a comprehensive materials characterization. The MoS_2_ QDs were added to the FAPI precursor solution in the concentrations of 0.5 mg mL^−1^, 1 mg mL^−1^ and 1.5 mg mL^−1^ and detailed FAPI and FAPI/MoS_2_ QDs film properties are presented. The charge carrier transport has been studied by evaluating the energy band diagrams (EBDs). We demonstrate that FAPI and MoS_2_ QDs form a type-I alignment that allows us to demonstrate the proposed type-I device architecture. We therefore report enhanced performance of FAPI/MoS_2_ QDs solar cells. The highest power conversion efficiency (PCE) of 15.01% was achieved with 1 mg mL^−1^ MoS_2_ QDs in FAPI film compared to PCE of 12.59% in the case of FAPI-only device.

## Results and discussion

### Material characterisation and energy band diagram evaluation

Here we refer to the different films as FAPI (FAPI only without MoS_2_ QDs), FAPI 0.5 (with 0.5 mg mL^−1^ MoS_2_ QDs), FAPI 1 (with 1 mg mL^−1^ MoS_2_ QDs) and FAPI 1.5 (with 1.5 mg mL^−1^ MoS_2_ QDs). The morphology of the films with the MoS_2_ QDs, see Fig. S2b–d in the supporting information (SI), appear more compact and homogenous compared to the FAPI only films (Fig. S2a in SI) with clearer and large grains. In the literature, it has been reported that MoS_2_ QDs assists in enhanced crystallinity of perovskite films and passivated interfacial defects^29^. The thickness of the films was examined by SEM from the films cross-sections (Fig. S3 in SI), which reported very similar values for all the films at the different MoS_2_ QDs concentrations. Average thickness values were 771 nm, 740 nm, 766 nm and 659 nm for the FAPI, FAPI 0.5, FAPI 1 and FAPI 1.5 respectively.

**Fig. 1:**
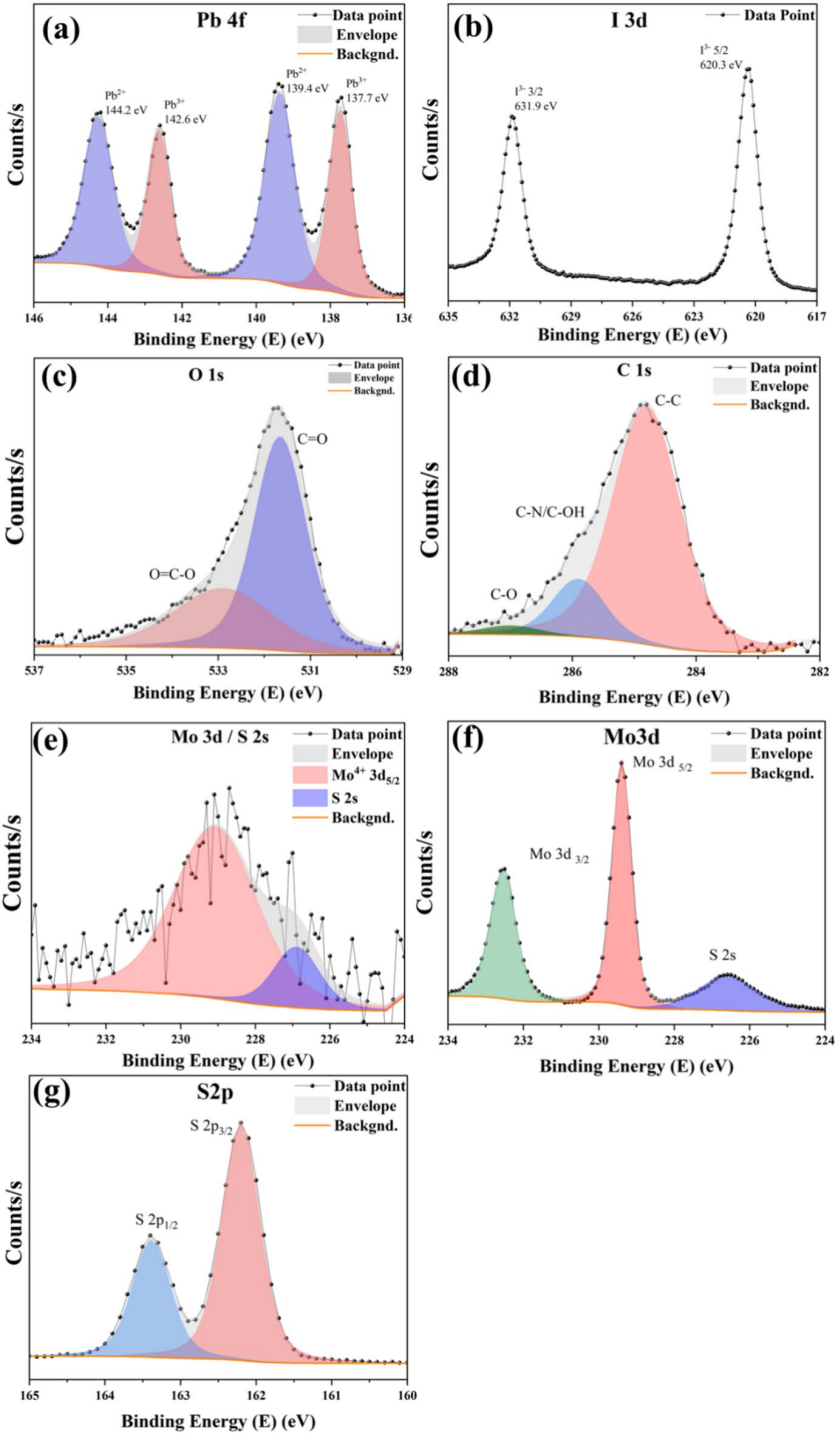
XPS spectrums for FAPI 1.5 film showing (**a**) Pb 4f showing Pb^2+^and Pb^3+^(FAPI) peaks, (**b**) I 3d orbital showing FAPI related peaks, (**c**) O 1 s orbital, (**d**) C 1 s orbital and (**e**) Mo 3d/S 2 s orbital. The XPS spectrums for drop-casted MoS_2_ QDs showing (**f**) Mo 3d orbital and (**g**) S 2p orbitals.

Further detailed X-ray photoelectron spectroscopy (XPS) analysis was conducted on the FAPI 1.5 film to examine the chemical composition of the FAPI/MoS_2_ film. FAPI 1.5 was chosen as it contains highest amount of MoS_2_. The peaks corresponding to Pb 4f and I 3d as well as those corresponding to O 1s, C 1s and N 1s were analysed (Fig. 1) as related to the chemical formula for FAPI, CH_3_(NH_2_)_2_PbI_3_^33^. Deconvolution of the peaks corresponding to Pb 4f_7/2_ and Pb 4f_5/2_ orbitals (Fig. 1a) indicated the presence of Pb^2+^ and Pb^3+^ oxidation states. The Pb 4f_7/2_ and Pb 4f_5/2_ spin-orbital peaks corresponding to Pb^3+^ are located at 137.7 eV and 142.6 eV, respectively (Fig. 1a). In the literature, these peaks are associated to δ-FAPI phase^34^. The I 3d orbital spectrum (Fig. 1b) shows I 3d_5/2_ and I 3d_3/2_ spin-orbitals at 620.3 eV and 631.9 eV respectively^34^. The Pb^3+^ peaks from Pb 4f orbital spectrum and I 3d orbital peaks are characteristic peaks for FAPI^35,36^. The Pb 4f_7/2_ and Pb 4f_5/2_ spin-orbital peaks, corresponding Pb^2+^ oxidation state are located at 139.4 eV and 144.2 eV respectively (Fig. 1a). These peaks indicate the formation of Pb(CO_3_)_2_ due to the adsorption of atmospheric CO_2_^37,38^. Formation of Pb(CO_3_)_2_ is also corroborated from the deconvolution of O 1s (Fig. 1c) and C 1s (Fig. 1d). The deconvolution of O 1s orbital spectrum indicates peaks corresponding to C=O (531.7 eV) and O=C–O (533.1 eV)^39^. Deconvolution of C 1s orbital spectrum shows peaks corresponding to C–C (284.8 eV) adventitious (aliphatic) carbon, C–N/C–OH (286 eV) and C–O (287.1 eV)^39^.

The presence of MoS_2_ QDs in the FAPI 1.5 film was confirmed by Mo 3d orbital spectrum (Fig. 1e) obtained from conducting XPS on the films. The deconvolution of Mo 3d spectrum reveals the presence of Mo^4+^ oxidation state as referred to Mo 3d_5/2_ spin-orbital peak located at 228.29 eV and S 2s spin orbital peak located at 226.31 eV. These peaks are characteristic peaks for MoS_2_^33^. For comparison, XPS was performed also on drop-casted MoS_2_ QDs on a gold-coated silicon wafer from a colloid in dimethylformamide (DMF). The gold layer was grounded with the XPS stage. Deconvolution of the Mo 3d peak shows Mo 3d_5/2_ (229.39 eV), Mo 3d_3/2_ (232.54 eV) and S 2s (226.60 eV) peaks related to Mo–S bonds (Fig. 1f)^40–42^. Similarly as shown in Fig. 1g, the doublet S 2p_3/2_ at 162.20 eV and S 2p_1/2_ at 163.39 eV represent Mo–S bonds^43,44^. These results confirm the chemical composition and stability of the MoS_2_ QDs without an oxide or a metallic component.

**Fig. 2:**
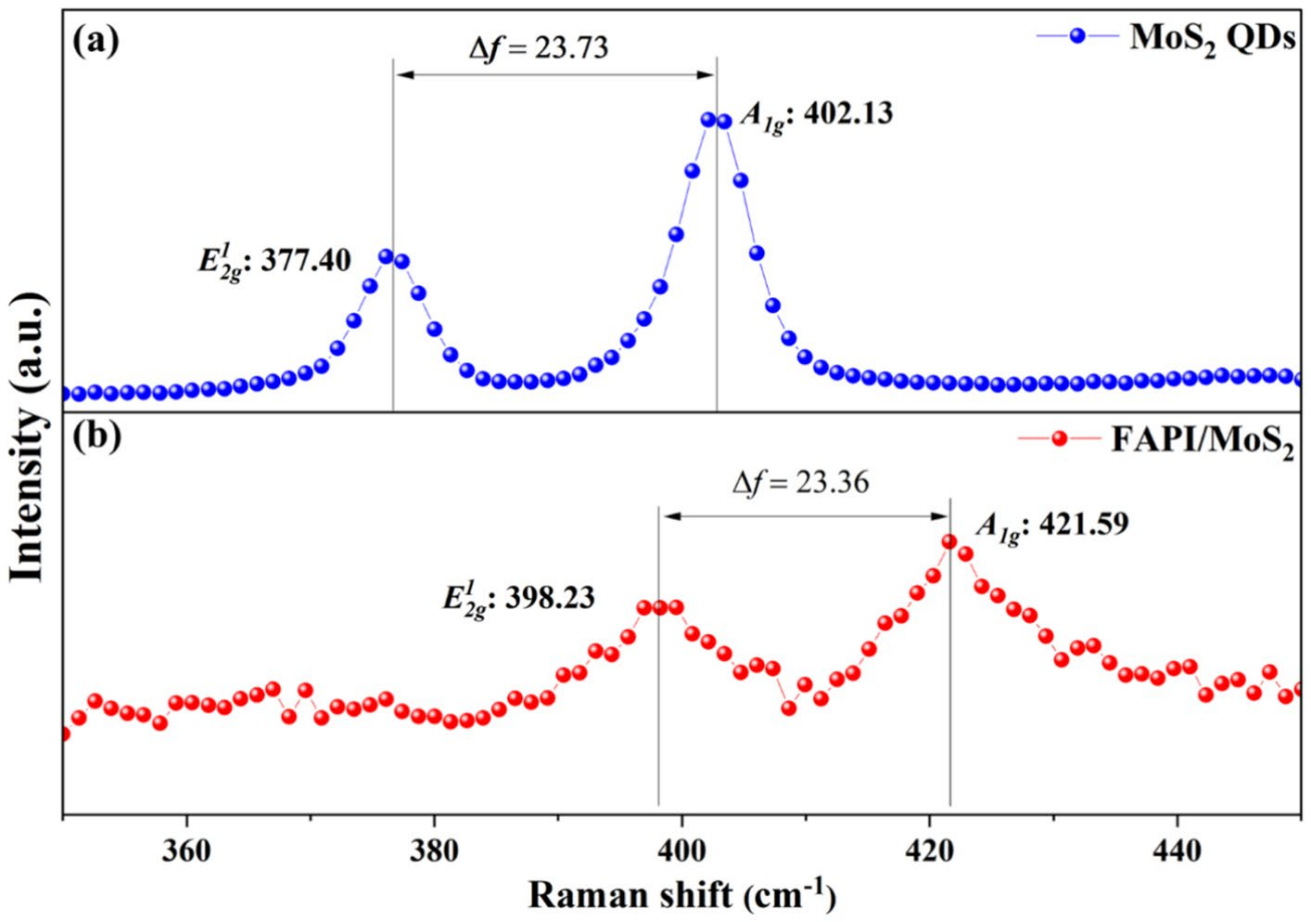
Raman spectrum of the (**a**) MoS_2_ QDs and (**b**) FAPI 1.5 film with MoS_2_ QDs related vibrational peaks.

The Raman spectrum of MoS_2_ QDs, Fig. 2a, shows the E_2g_^1^ mode (in-plane vibrations) at 377.40 cm^−1^ and A_1g_ mode (out-of-plane vibrations) at 402.13 cm^−1^. The Raman spectrum corresponding to FAPI 1.5 film (Fig. 2b) shows the presence of E_2g_^1^ mode at 398.23 cm^−1^ and A_1g_ mode at 421.59 cm^−1^. The difference (*Δf*) between Raman modes is *Δf* = 23.73 cm^−1^ for MoS_2_ QDs and *Δf* = 23.36 cm^−1^ for FAPI 1.5 film. In the literature, *Δf* values of 24.4 cm^−1^, 25.7 cm^−1^ and 25.9 cm^−1^ are reported for MoS_2_ QDs^45–47^. The *Δf* value provides an estimate of the number of layers in MoS_2_ QDs, with higher values indicating fewer number of layers as Fewer layers result in less-stiffening of the vibrations. In the case of our work, the *Δf* value for FAPI 1.5 film is lower as compared to only MoS_2_ QDs. This indicates stiffening of both out-of-plane and in-plane vibrations in FAPI 1.5 film, which is reasonable as MoS_2_ QDs are embedded into the FAPI matrix. Similarly, this effect can also be noticed from the red-shift of the individual E_2g_^1^ and A_1g_ modes in the case of FAPI 1.5 film. The red-shift has been also reported to be associated with stiffening of the Mo-S bond vibrations^46,47^.

**Fig. 3:**
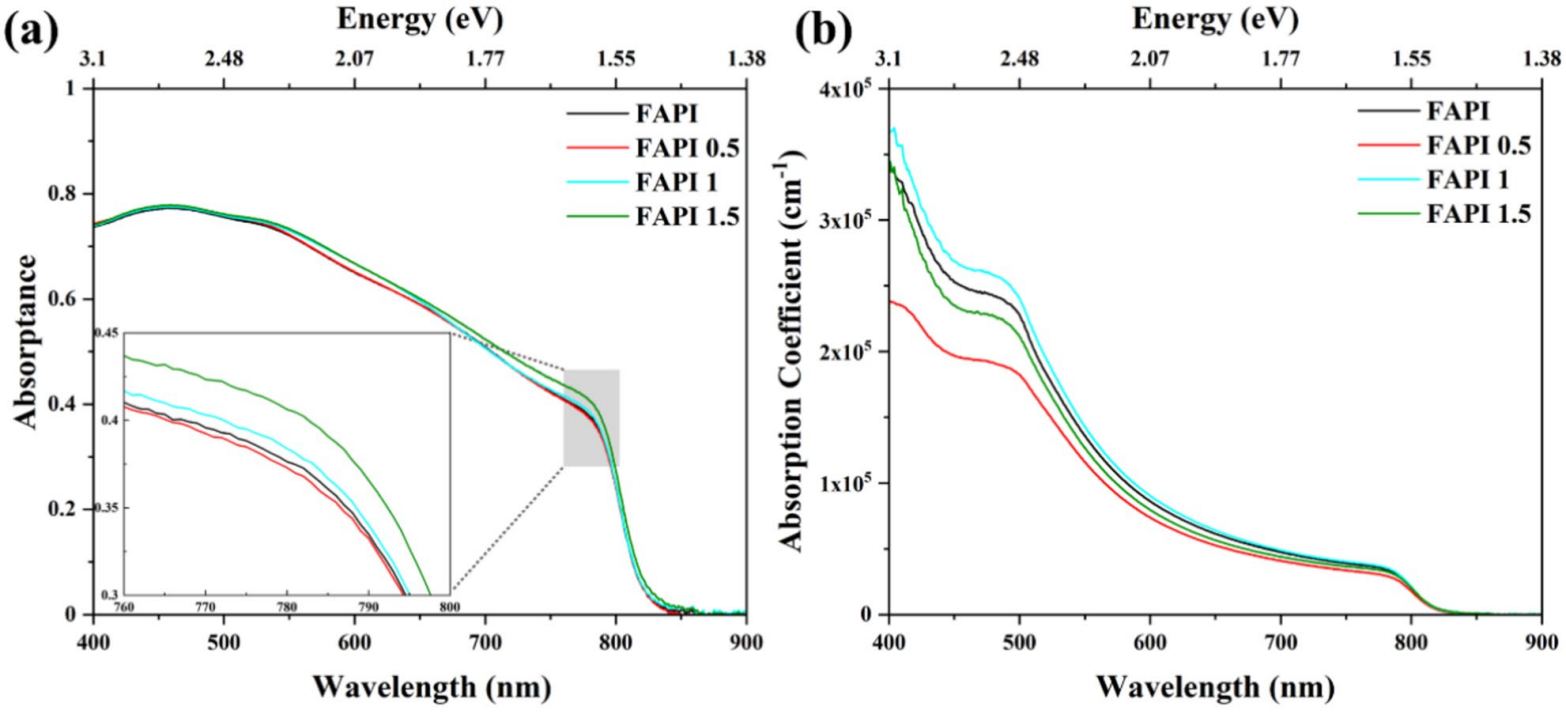
Optical characterisation from UV–Vis measurements of FAPI, FAPI 0.5, FAPI 1 and FAPI 1.5 films showing (**a**) absorptance and **(b**) absorption coefficient.

The optical properties were examined by analysing absorptance and absorption coefficient of the films. It should be noted that the measurements are impacted due to fast degradation of the films. As shown in Fig. 3a, absorptance is in the range of 400–800 nm which is consinstent with the characteristics of good quality FAPI based films^48^. FAPI 0.5 film shows slightly lower absorptance compared to the FAPI-only film, However, FAPI 1 and FAPI 1.5 show an increase in absorptance compared to FAPI-only. Due to the low concentration of MoS_2_, the difference is relatively small compared to FAPI-only and among the films with different MoS_2_ concentrations. The absorption coefficient of the films was calculated with respect to film thicknesses. The film thicknesses were evaluated by a profilometer on different spots on each film (Fig. S4 in SI) and the median value was taken as the final thickness. The thickness of the individual films used for optical characterization was found to be 210 nm, 200 nm, 200 nm, 210 nm for FAPI, FAPI 0.5, FAPI 1 and FAPI 1.5 respectively. The highest absorption coefficient was obtained from FAPI 1 film. The absorption coefficient is of the order of 10^5^ cm^−1^, as shown in Fig. 3b, suggesting that 1 mg mL^−1^ of MoS_2_ QDs concentration is optimal for FAPI-based devices.

The proposed type-I alignment in this work crucially hinges on the position of energy levels of the two materials i.e. FAPI and MoS_2_ QDs. The wide bandgap material (MoS_2_ QDs) should align with the narrower bandgap material such that photogenerated carriers (electrons and holes) are transported from the MoS_2_ QDs to the FAPI layer. To obtain the bandgap, Tauc plots were constructed on the basis of ultraviolet–visible transmittance and reflectance measurement results. In the case of the composite films, direct transition bandgap was observed at 1.533 eV, 1.533 eV, 1.533 eV and 1.531 eV for FAPI, FAPI 0.5, FAPI 1 and FAPI 1.5 respectively (Fig. S6a in SI). These are all very close to the value of 1.53 eV, confirming that the inclusion of MoS_2_ QDs has not caused disruption to the FAPI film. For MoS_2_ QDs, the direct transition bandgap of 1.63 eV was observed (Fig. S6b in SI). These values are suitable to produce a type-I alignment, whereby the bandgap of the FAPI film could be nested within the larger bandgap of the MoS_2_ QDs.

The valence band maximum (VBM) and Fermi level (E_f_) values for MoS_2_ QDs were measured by ultraviolet photoelectron spectroscopy (UPS). The UPS spectrum obtained from MoS_2_ QDs is presented in Fig. S5 of the SI. The E_f_ level was determined from the cut-off edge of the secondary electrons at 16.45 eV, returning the E_f_ value of − 4.77 eV. The onset of the spectrum is located at 1.06 V, which returns the difference between VBM and E_f_. Therefore, using the E_f_ value obtained, the VBM energy level is estimated at − 5.83 eV. The conduction band minimum (CBM) value of the MoS_2_ QDs was then calculated from the measurements of the VBM and bandgap, which produced a value of − 4.2 eV (− 5.83 eV + 1.63 eV). Table 1 shows the summary of the energy band levels for FAPI and MoS_2_ QDs (values for FAPI were taken from the literature).

**Table 1:** Summary of energy band values of the FAPI film and MoS2 QDs.

Sample	E_f_ (eV)	VBE (eV)	E_g_ (eV)	CBE (eV)
FAPI	− 4.4	− 5.4	1.4	− 4
MoS_2_ QDs	− 4.77	− 5.83	1.63	− 4.2

**Fig. 4:**
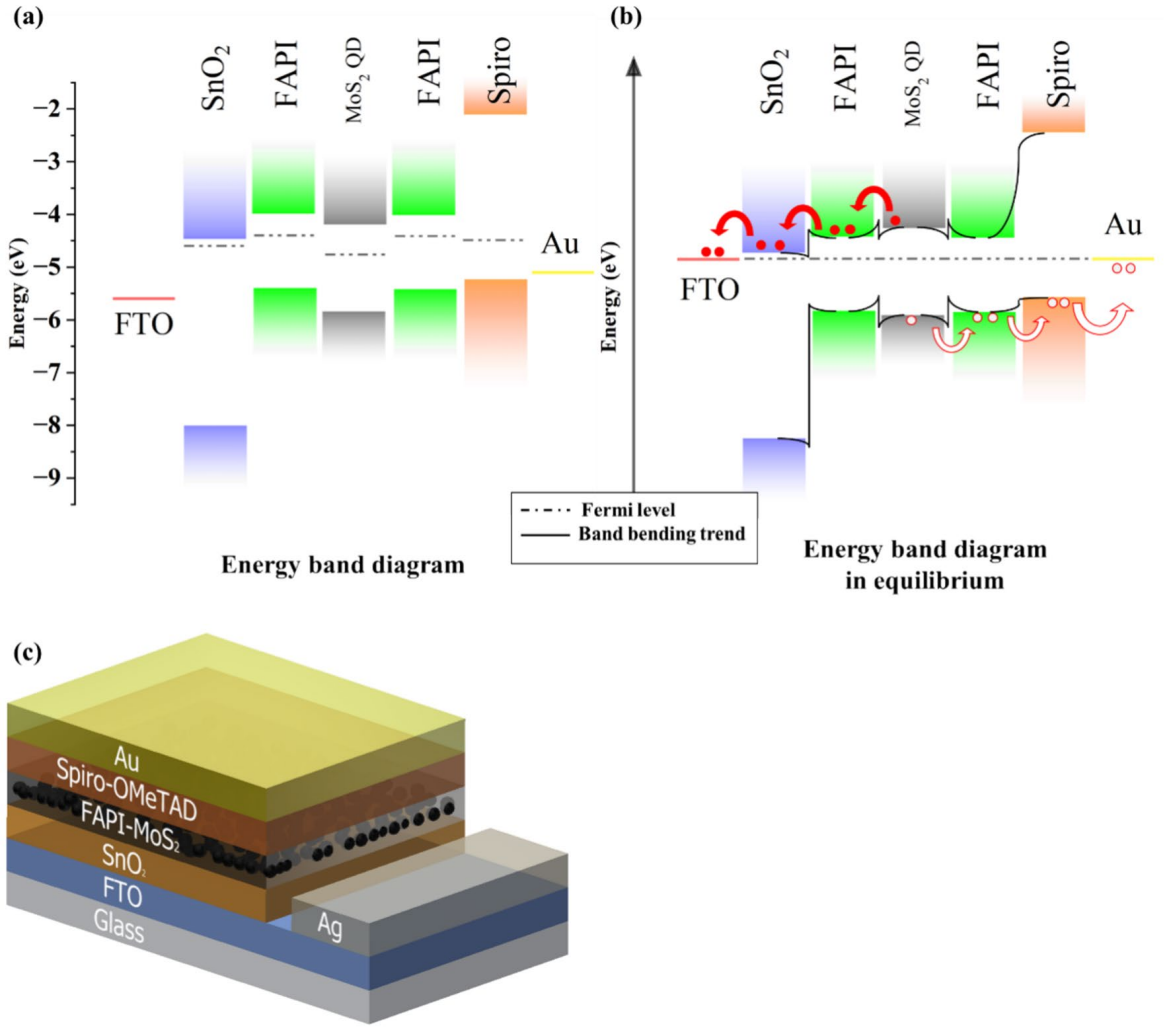
(**a**) Energy band alignment diagram (EBD) for type-I solar cell with FAPI/MoS_2_ as active layer, tin oxide (SnO_2_) as electron transport layer and spiro-OmeTAD as hole transport layer. (**b**) EBD in equilibrium.

Based on the energy band positions for FAPI and MoS_2_ QDs, Fig. 4a,b shows the energy band diagram (EBD) for FAPI and MoS_2_ QDs separately and in equilibrium. A small energy barrier is formed due to the mismatch between Fermi levels. However, these barriers are relatively small and with reduced impact on carrier transport when the absorber layer operates in full depletion under an electric field. Hence good adherence of type-I band alignment is observed between the MoS_2_ QDs and FAPI.

### Solar cell architecture and performance

The photovoltaic (PV) architecture was evaluated with SnO_2_ as the electron transport layer (ETL), Spiro as the hole transport layer (HTL) and FAPI (or FAPI/MoS_2_ QDs) as the absorber layer. Figure 4a,b shows the EBD of the proposed PV architecture of the FAPI/MoS_2_ film with all the layers involved.

Figure 4c shows the schematic of the device fabricated on fluorine-doped tin oxide (FTO) coated glass. SnO_2_ as ETL, FAPI/MoS_2_ film as the absorber layer, spiro as the HTL and Au as the metal contact. The thicknesses of the individual layers in the device were evaluated by scanning electron microscopy (SEM) on the cross-section of the device (Fig. S7b). The thicknesses measured from SEM image for FTO, SnO_2_, FAPI/MoS_2_ QDs, spiro and Au are 100 nm, 400 nm, 750 nm, 300 nm and 200 nm, respectively. Energy dispersive X-ray spectroscopy (EDS) maps were obtained for each layer to investigate the chemical composition shown in the SI (Fig. S7d–m). Distinct colour maps are observed for each layer. Furthermore, the EDS line scan was conducted across the device layers, shown in the SI (Fig. S7n). The thicknesses for the individual layers were also obtained, which corroborate well with thicknesses obtained from cross-sectional SEM.

**Fig. 5:**
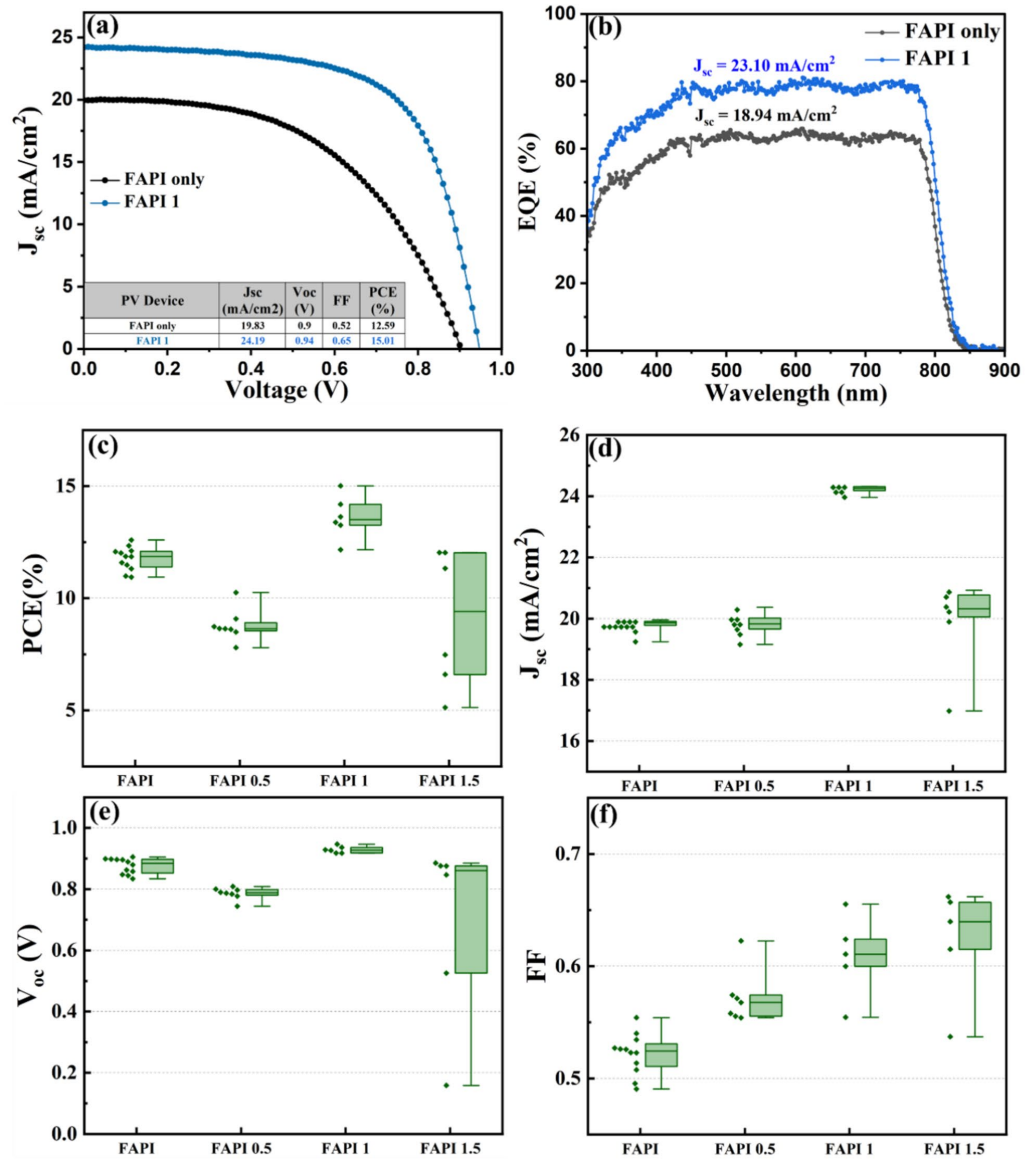
Solar cell performance with type-I device architecture FTO-SnO_2_-perovskite-spiro-Au. (**a**) Shows the J–V characteristics of champion devices with FAPI and FAPI 1 active layer. (**b**) EQE of the FAPI and FAPI 1 device. Statistics of performance parameters (**c**) short-circuit current density (J_sc_), (**d**) open-circuit voltage (V_oc_), (**e**) fill factor and (**f**) photo conversion efficiency (PCE).

PV devices were fabricated with FAPI only, FAPI 0.5, FAPI 1 and FAPI 1.5 films as the absorber layers. Performance of these devices were evaluated by current density–voltage (J–V) characterisation. The active surface area of our devices was ~ 23 mm^2^ and a shadow mask was used during illumination. The best performance was observed with the FAPI 1 film, i.e. with 1 mg mL^−1^ MoS_2_ QD in FAPI. Figure 5a shows the comparison between the champion devices of FAPI only and FAPI 1.

We observed that the FAPI 1 champion device exhibits the highest power conversion efficiency (PCE) of 15.01% as compared to the FAPI-only champion device which exhibits the efficiency of 12.59%. The corresponding devices resulted in J_SC_, V_OC_ and fill factor (FF) of 24.19 mA cm^−2^, 0.94 V and 0.65 respectively in case of FAPI 1 device. FAPI-only devices resulted in J_SC_, V_OC_ and FF of 19.83 mA cm^−2^, 0.9 V and 0.52 respectively. The rise in efficiency in the devices, with the inclusion of MoS_2_ QDS, could be attributed to enhanced generation and transport of the photogenerated carriers from the MoS_2_ QDs and due to type-I architecture, in addition to the photogenerated carriers from FAPI film. Consistently to the J–V curve (Fig. 5a), the external quantum efficiency (EQE, Fig. 5b) of FAPI 1 shows the maximum PCE of around 80% for incident wavelengths in the range of 400–800 nm as compared to 60% for the FAPI only. Unfortunately, as the bandgap value of the two components are very close to each other (1.53 eV for FAPI and 1.63 eV for MoS_2_ QDs), it is not possible to clearly see the change in slope of the EQE signal. Corresponding current density calculated from EQE measurements for FAPI only and FAPI 1 devices are 18.94 mA cm^−2^ and 23.10 mA cm^−2^, which are sufficiently close to the values obtained from J–V characteristics. Statistics of performance parameters for FAPI only, FAPI 0.5, FAPI 1 and FAPI 1.5 are shown in Fig. 5c–f. The FAPI 0.5 and FAPI 1.5 show relatively low performance overall, confirming the optimal concentration of MoS_2_ QDs at 1 mg mL^−1^, whereby different concentrations might either disrupt transport (high concentration) or produce an insufficient contribution to the absorption (low concentration, see also Fig. 3b).

In order to understand the contribution of MoS_2_ QDs in the photocurrent we analysed further the EQE measurements. The minority carrier diffusion length (*L*_*diff*_) was calculated for the two different devices from the data points of the EQE in the range 800–850 nm. Figure S8a in the SI shows the x-intercept of IQE^−1^ vs α^−1^ which gives the *L*_*diff*_ values for FAPI and FAPI 1 of 0.325 µm and 0.753 µm. The higher minority carrier diffusion length may be attributed to FAPI interface defect passivation and reduction of grain boundaries (e.g. see Fig. S2 in SI) as a result of the MoS_2_ QDs inclusion^29^. The inclusion of MoS_2_ QDs in FAPI shows more than doubling of the *L*_*diff*_ values, which confirms that the type-I architecture can contribute to improved photogenerated carrier transport. The performance enhancement due to type-I architecture is also corroborated from the J–V dark current analysis, shown in the SI, Fig. S8b. As seen from the space charge region, the device corresponding to FAPI 1 shows higher J_SC_ in the diffusion region indicating qualitatively higher *L*_*eff*_. Similarly, the J_SC_ values in recombination region for FAPI 1 devices is lower compared to FAPI-only indicating lower recombination rates.

## Conclusion

We have successfully produced stable and crystalline composite films of FAPI with MoS_2_ QDs. SEM images revealed that the presence of MoS_2_ resulted in smaller grains, augmenting the mechanical robustness of the films. The presence of MoS_2_ QDs in FAPI was verified through XPS and Raman spectra and the analysis of the energy band levels reveals a type-I alignment. The use of composite FAPI/MoS_2_ QDs films resulted in an enhancement in the efficiency of corresponding devices from 11.76 to 13.61% (average values from statistical analysis). The comparison of carrier diffusion length in FAPI and FAPI/MoS_2_ QDs films indicated a higher diffusion length in FAPI/MoS_2_ QDs, suggesting that MoS_2_ QDs not only contribute to the passivation of interface defects but also improve the transport of photogenerated carriers due to the type-I architecture. Our results demonstrate the feasibility of devices with a type-I architecture, which could benefit from synergies of different materials and provide a realistic avenue for quantum dot solar cells, overcoming the long-standing challenge of carrier transport.

## Experimental details

### Chemicals and materials

Formamidinium iodide (CH_5_IN_2_, purity ≥ 99% anhydrous), methylammonium chloride (CH_6_ClN, purity ≥ 99.0%) and lead (II) iodide (PbI_2_, purity 99.999% trace metals basis) were purchased from Sigma-Aldrich and used as precursors to produce the FAPI films. Dry molybdenum sulphide (MoS_2_) QDs powder was synthesised following a previous reported recipe and procedures^47^. Dimethylformamide (DMF, purity > 99%) and dimethyl sulfoxide (DMSO, purity > 99%) was purchased from Sigma-Aldrich. Tin (IV) oxide (SnO_2_) colloid was purchased from Alfa Aesar, 15% wt in de-ionized (DI) water. Titanium diisopropoxide bis(acetylacetonate), titania paste, butanol (anhydrous, 99.8%), ethanol (anhydrous, ≥ 99.5%). 2,2′,7,7′-tetrakis-(N,N-di-4-methoxyphenylamino)-9,9′ spirobifluorene (Spiro-OMeTAD), 4-*tert*-butypyridine(tBP), lithium bis(trifluoromethanesulfonyl)imide(Li-TFSI), isopropyl alcohol and n-octlyammonium iodide (OAI) were purchased from Sigma-Aldrich. 15 MΩ cm de-ionized (DI) water is used throughout the process. Fluorine-doped tin oxide (FTO) coated glass substrates were purchased from Visionteksystems. MoS_2_ QDs powder is prepared by synthesis process detail in literature^47^.

### Characterisation techniques

Scanning electron microscopy (SEM) was conducted using Hitachi SU5000 microscope. The electron gun was operated at 15 kV under high-vacuum condition. Energy dispersive X-ray spectroscopy (EDS) was performed by an in-situ Oxford Instruments X-max 80 detector. X-ray photoemission spectroscopy (XPS) was performed using an ESCALAB 250 Xi spectrometer (Thermo Fisher Scientific, UK) using a monochromated aluminium anode with an excitation energy of 1486.68 eV. The analysis chamber pressure was maintained at 1 × 10^−9^ mbar. The samples were prepared by spin coating the FAPI precursor solution on quartz substrate. Charge correction was done against the platinum (Pt) peak at 71.2 eV corresponding to 4f orbital. Pt foil was loaded in the XPS with every sample and was grounded with the stage. The same equipment chamber and detector was used for ultraviolet photoemission spectroscopy (UPS) however with a helium discharge source (He I: 21.2 eV) as excitation to determine the fermi-level (*E*_*f*_) and the valence band maximum (VBM). A negative bias of − 10 V was applied to the stage during the measurements. During XPS and UPS analysis the surface of the sample was grounded with the equipment stage. Raman spectroscopy was performed with an inVia™ Qontor™ confocal Raman microscope with 532 nm laser. Ultraviolet–visible spectroscopy (UV–Vis) was performed using a PerkinElmer Lambda 1050^+^ UV–Vis spectrophotometer with the spectral range of 190–3500 nm. The monochromator was set at 1 nm step size. The equipment was fitted with 150 mm integrating sphere for transmittance and reflectance measurements. The samples for UV–Vis spectroscopy were prepared by spin coating the FAPI precursor solution on quartz substrates. Transmittance (*T*) was obtained by placing the sample in the transmittance port (front-end) of the integrating sphere. Reflectance was measured by placing the sample at reflectance port (back-end on the integrating sphere). The calculation of absorptance, absorption coefficient and bandgap from *T* and *R* spectra of the films are described in the supporting information (SI, Section S1). The optical properties of MoS_2_ QDs were evaluated by performing UV–Vis on colloidal samples of MoS_2_ QDs in DMF. Transmittance was obtained by placing the cuvette at the transmittance port. The combined signal of transmittance and scattering (*T* + *S*) was obtained by placing the cuvette at the centre of the integrating sphere.

The performance of solar cells was tested under AM1.5G solar spectrum with irradiance of 100 mW cm^−2^ using Wacom Electric Co. solar simulator (JIS, IEC standard con- forming, CLASS AAA). An aperture mask of 0.2275 cm^2^ was used to incident the light on the devices. The spectral response was calibrated using an a-Si reference cell. Keithley 2400 source meter was used to record electrical data. The external quantum efficiency (EQE) of the solar cells was measured using an Eko Seiki SPM-005B system in direct current (DC) mode with 150 W Xenon lamp. A a-Si reference cell was used to calibrate the spectral response also in this case. The current density (*J*_*sc*_) and the minority carrier diffusion length (*L*_*diff*_) were calculated using the EQE data (see SI, Section S2).

### Solar cell fabrication

The step-by-step schematic diagram of the solar cell fabrication process is reported in the SI (Fig. S1). Devices were fabricated on glass substrates coated with fluorine-doped tin oxide (FTO). The substrates were cleaned by sonication in four different solutions for 10 min each in the following order: 2% Hellmanex III solution in DI water, DI water, acetone and ethanol. Substrates then were dried with a nitrogen gun and exposed to 20 min ozone using photo surface processor from SEN LIGHTS Corporation, model pl16-110.

SnO_2_ was used as the electron transport layer (ETL). The SnO_2_ colloid is diluted by 1:1 volume ratio in water, followed by filtration with 0.22 µm filters. 300 µL of this solution was then spin coated at 3000 rpm for 30 s followed by annealing in air at 150 °C for 30 min. Samples were allowed to cool down to ambient temperature following this and any other annealing process. The substrates with ETL were then treated with ozone for 10 min before depositing the next layer. The deposition of the absorber layer, FAPI without MoS_2_ QDs, was carried out in a nitrogen-ventilated glove box where the relative humidity was less than 10%. The precursor solution was prepared by dissolving PbI_2_ (1.92 M), CH_6_ClN (0.64 M) and CH_5_IN_2_ (1.83 M) in a 4:1 ratio by volume of DMF and DMSO. In order to prepare the composite absorber layer of FAPI with the MoS_2_ QDs, different amounts of the MoS_2_ QDs (1.5 mg, 1 mg and 0.5 mg) were dispersed in a solution of DMF and DMSO with a 4:1 ratio by volume. Subsequently, PbI_2_ (1.92 M), CH_6_ClN (0.64 M) and CH_5_IN_2_ (1.83 M) were added. The colloidal was left on the shaker overnight to thoroughly mix the added powders. The colloid or solution (50 µL) of the absorber layer was spin-coated at 4000 revolution per minute (rpm) for 30 s and 300 µL of antisolvent anisole was dropped from a ~ 0.7 cm diameter orifice at approximately 20 s in the spin-coating process^49^. The samples were then heated on a hotplate at 150 °C for 15 min. The film visibly crystallises within seconds to form a mirror like black textured finish.

Spiro-OMeTAD was used as the hole transport layer (HTL) and the deposition was continued in the glovebox. The precursor solution was prepared by dissolving 216 mg of Spiro-OMeTAD in chlorobenzene. A 52.5 µL mixture of 39 mg Li-TFSI in 75 µL acetonitrile and 85.5 µL was then added to the Spiro-OMeTAD precursor solution, which was then spin-coated in an N_2_-ventilated glove box where relative humidity was less than 10%. 300 µL of the solution was spin-coated at 4000 rpm for 30 s and was left to dry at room temperature. The devices fabricated and with Spiro-OMeTAD were then stored in a humidity controlled dry box overnight. Au-contacts are deposited on spiro by thermal evaporation of gold wire followed by application of an Ag paste on the underlying FTO layer to form Ag contacts. The total active device surface area was ~ 23 mm^2^ and an aperture mask of 0.2275 cm^2^ was used to characterise the devices. The devices were stored in the dry box overnight to allow Ag paste to solidify.

## Supplementary Information

Supplementary Information.

## Data Availability

This paper is accompanied by representative samples of experimental data and the relevant numerical tabulated raw data is available from the University of Strathclyde’s Research Portal at 10.15129/864c99bb-a8d0-4528-9ea0-28b0396675a4. Detailed procedures explaining how these representative samples were selected, and how these experiments can be repeated, are provided in the corresponding sections of this paper. Additional results and raw data underlying this work are available in the Supporting Information or on request following instructions provided at 10.15129/864c99bb-a8d0-4528-9ea0-28b0396675a4.
