# Supplementary material for: Formamidinium lead iodide perovskite photovoltaics with MoS2 quantum dots
Source: Sci Rep. 2024 Sep 16;14:21613. doi: 10.1038/s41598-024-72037-3 (PMC11405757; doi:10.1038/s41598-024-72037-3)

**Supporting information**

**Formamidinium lead iodide perovskite photovoltaics with MoS2 quantum dots**

Ankur Uttam Kambley,^*²^ Bruno Alessi,^§^ Calum McDonald,^§^ Pagona Papakonstantinou,^*^ Vladimir Svrcek,^§²^ Davide Mariotti ^Ϯ²^

* School of Engineering, Ulster University, York Street, Belfast BT15 1ED, United Kingdom.

§ Renewable Energy Research Center, National Institute of Advanced Industrial Science and Technology (AIST), Tsukuba, Ibaraki 305-8568, Japan.

Ϯ Department of Design, Manufacturing & Engineering Management, University of Strathclyde, Glasgow, UK

^⯎^ Corresponding authors: [*kambley.a1@gmail.com*](mailto:kambley.a1@gmail.com); [*vladimir.svrcek@aist.go.jp*](mailto:vladimir.svrcek@aist.go.jp); *davide.mariotti@strath.ac.uk*

Absorptance, absorption coefficient and bandgap calculations for FAPI films:

|  | $Absorptance=1-(T+R)$ | (1) |
| --- | --- | --- |

The absorption coefficient (α) of the films was calculated by following equation:

|  | $\alpha=-\frac{1}{t}\left\{ ln\frac{T}{1-\left( T+R \right)+T} \right\}$ | (2) |
| --- | --- | --- |

Where,

t : thickness of the film in cm.

T: transmittance obtained from UV-Vis

R: reflectance obtained from UV-Vis

Furthermore, the direct-transition Tauc plot was calculated by following equation:

|  | $\left( \alpha.h\nu\right)^{2}=h\nu-E_{g}$ | (3) |
| --- | --- | --- |

Where,

α = absorption coefficient of the film

h = Plank’s constant

ν = photon’s frequency

E_g_ = band gap energy

Plotting the (α.hν)^2^ on y-axis and hν on x-axis and extrapolating the slope the to y = 0 gives hν = E_g_. Therefore the x-intercept extrapolating the linear slope gives out E_g_ value. In case of calculation of optical properties for MoS_2_ colloidal the “R” parameter is substituted with scattering values (S) since for colloids R<<S and *t* represents optical length of the cuvette.

The current density (*J_sc_*) of the devices was calculated, by the in-built software, from the EQE data by following equation:

|  | $J_{sc}=q\int\phi\left( \lambda\right) . EQE\left( \lambda\right) d\lambda$ | (4) |
| --- | --- | --- |

Where,

*q* : charge of the electron

ϕ : photon flux corresponding to AM1.5G spectrum

λ : Wavelength

To understand the diffusion length of the carriers in FAPI and FAPI/MoS_2_ films, minority carrier diffusion length calculations (L_diff_) were performed on the data points from trailing end (800 nm - 850 nm) of the EQE plot. The equation below shows the linear relation between parameters internal quantum efficiency and absorption coefficient of the active layer:

|  | ${IQE}^{-1}=1+\alpha^{-1}\frac{\cos\theta}{L_{diff}}$ | (5) |
| --- | --- | --- |

Where,

IQE: Internal quantum efficiency

α: Absorption coefficient of the active layer

L_diff_: minority carrier diffusion length

Extrapolating the data points to x-axis i.e. IQE^-1^ = 0, we get:

|  | $L_{diff}={-\alpha}^{-1}$ | (6) |
| --- | --- | --- |

IQE is calculated from the following equation:

|  | $IQE=\frac{EQE}{1-R}$ | (7) |
| --- | --- | --- |

Where,

R: Reflectance of the active layer


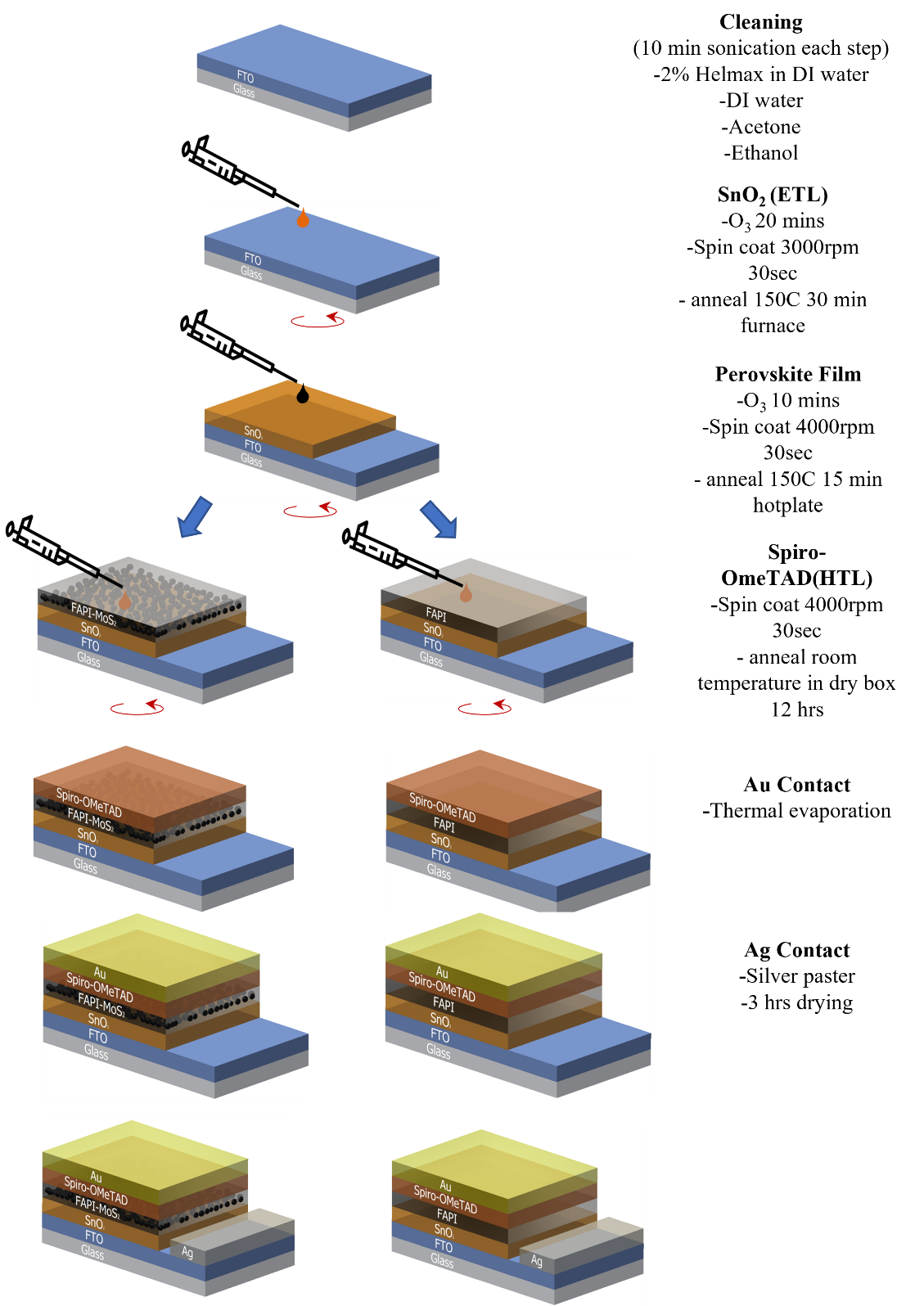


Figure S1: Schematic diagram of FAPI/MoS_2_ based solar cells.


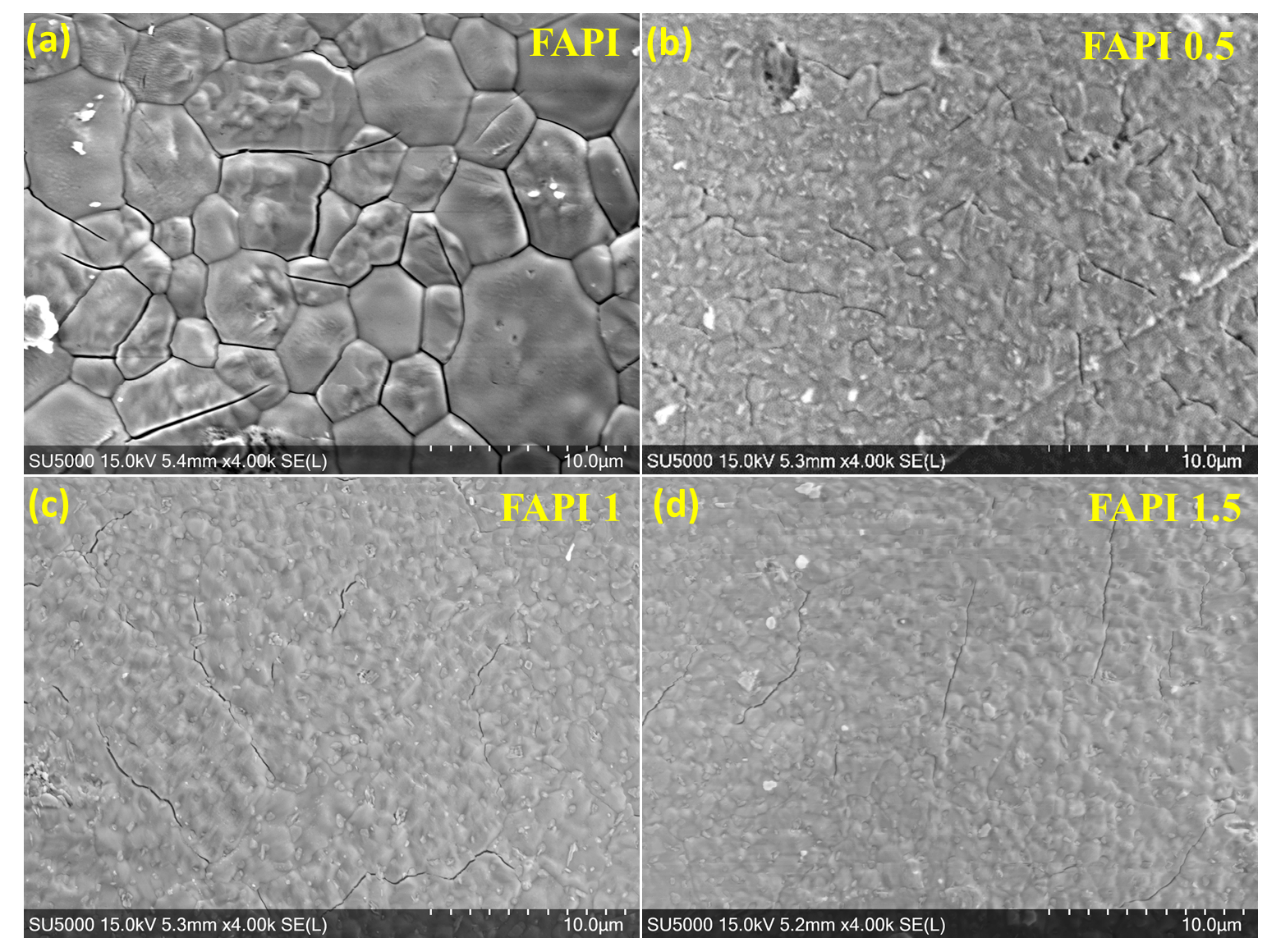


Figure S2: SEM top-view images of films (a) FAPI, (b) FAPI 0.5, (c) FAPI 1 and (d) FAPI 1.5.

Figure S3: SEM images of films (a) FAPI, (b) FAPI 0.5, (c) FAPI 1 and (d) FAPI 1.5. (e) Shows the film thicknesses evaluated from cross-sectional SEM image.


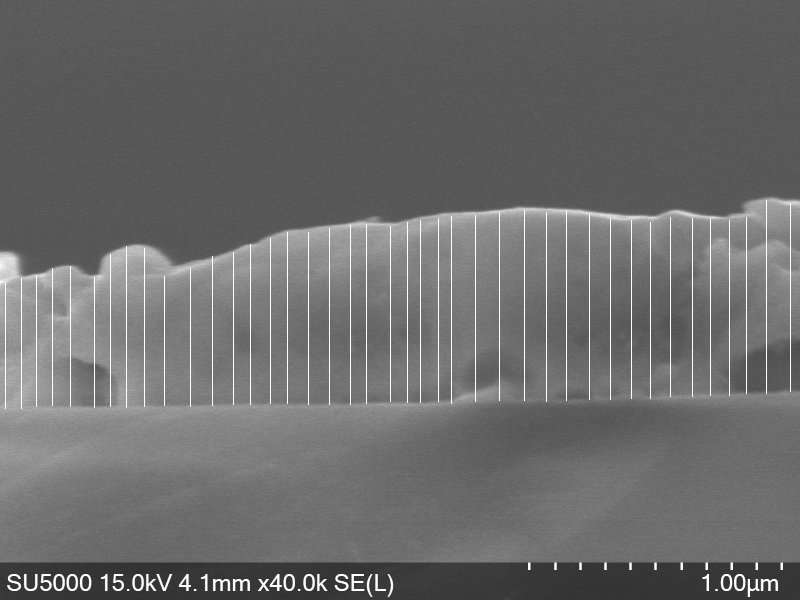


**FAPI 1.5**


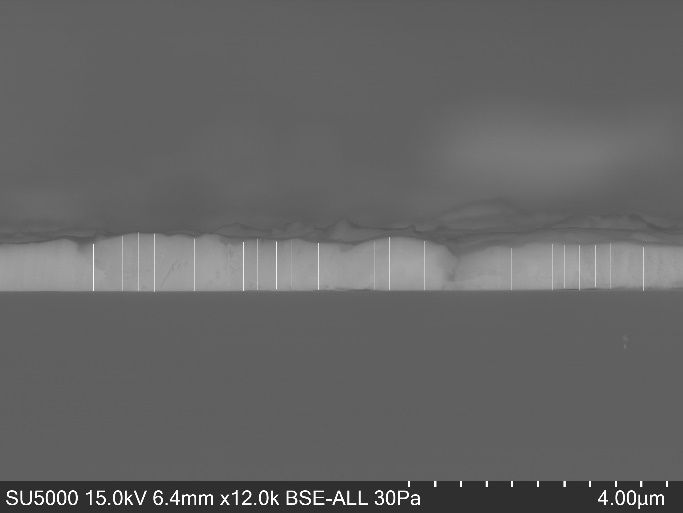


**FAPI**


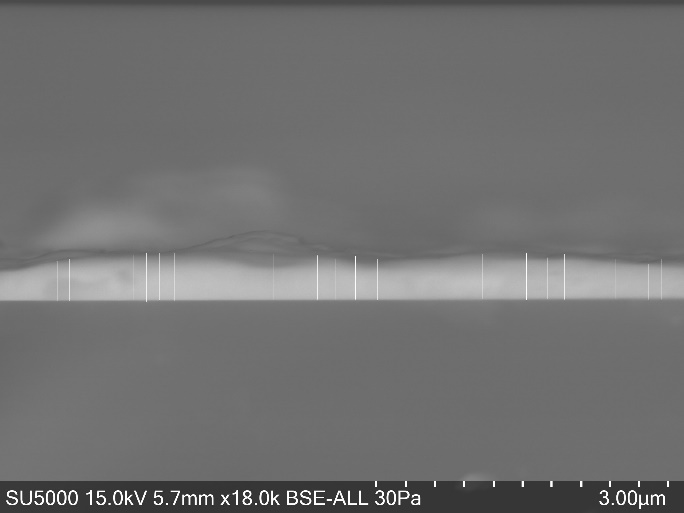


**FAPI 1**


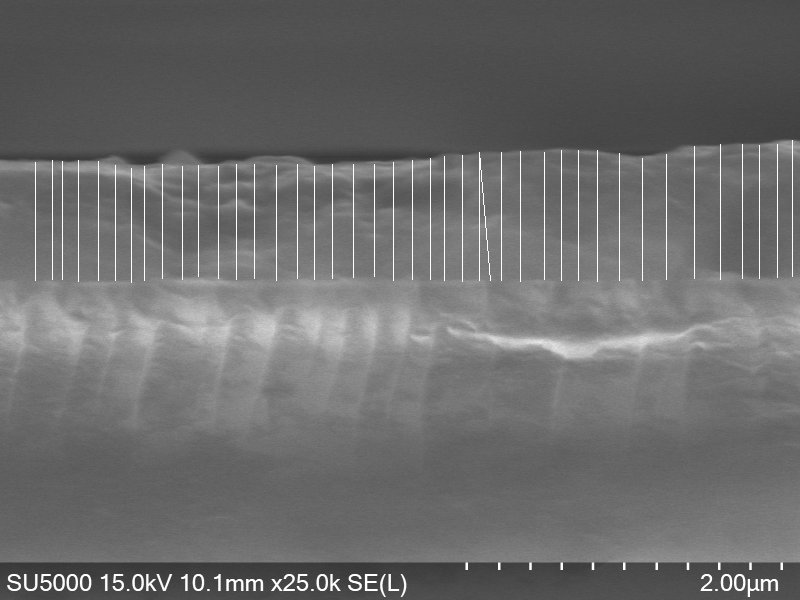


**FAPI 0.5**

**(a)**

**(b)**

**(c)**

**(d)**

**(e)**


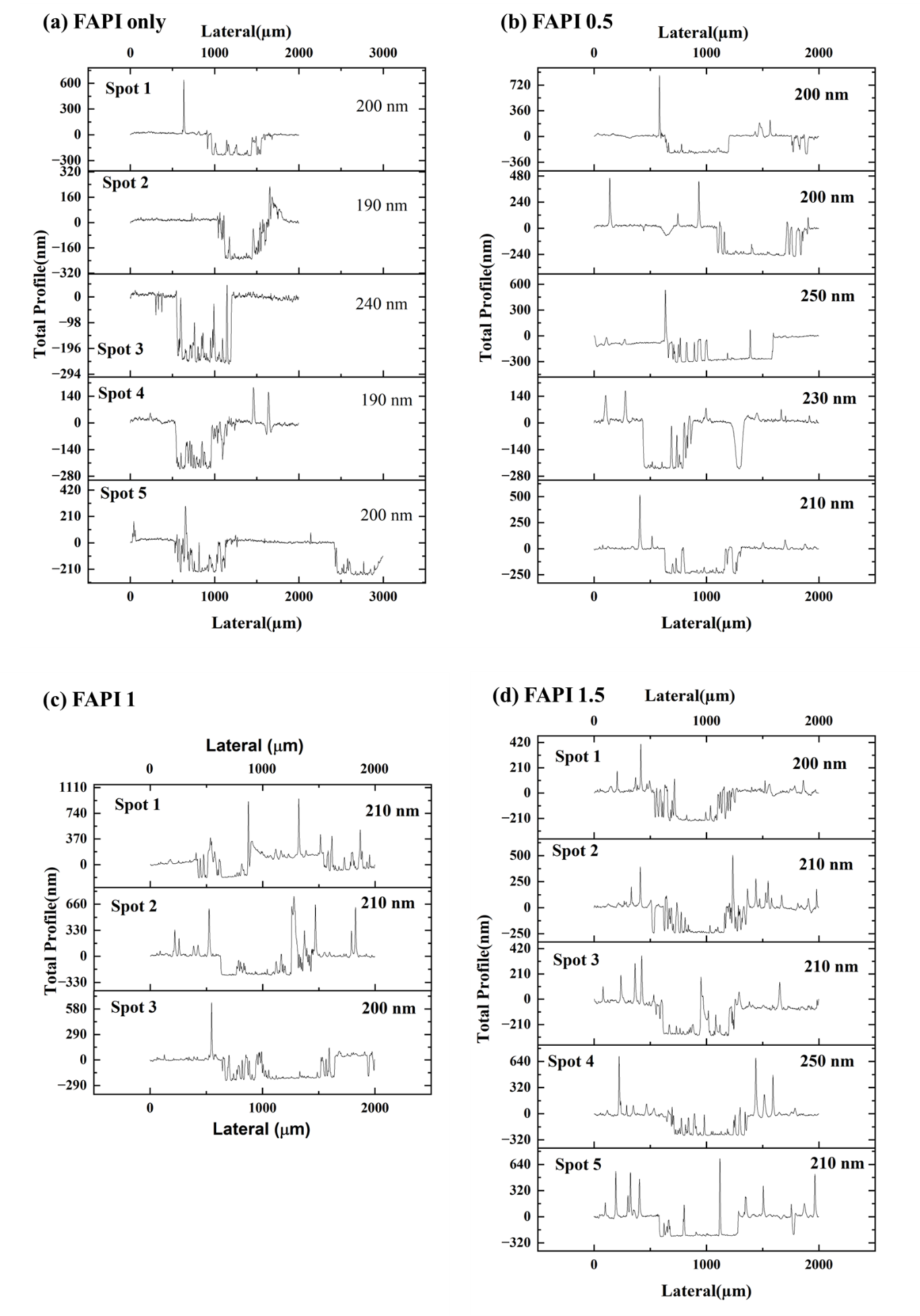


Figure S4: Film thickness results obtained from profilometer showing thickness of (a) FAPI only, (b) FAPI 0.5, (c) FAPI 1 and (d) FAPI 1.5.


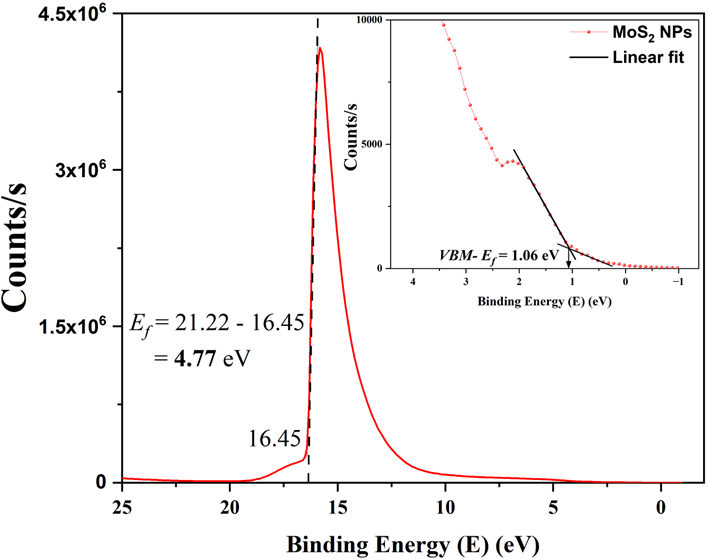


Figure S5: UPS spectrum of MoS_2_ NPs showing the fermi-level at 4.40 eV extrapolated from secondary electron cut-off and VBE-E_f_ of 1.02 extrapolated from on-set of the graph.


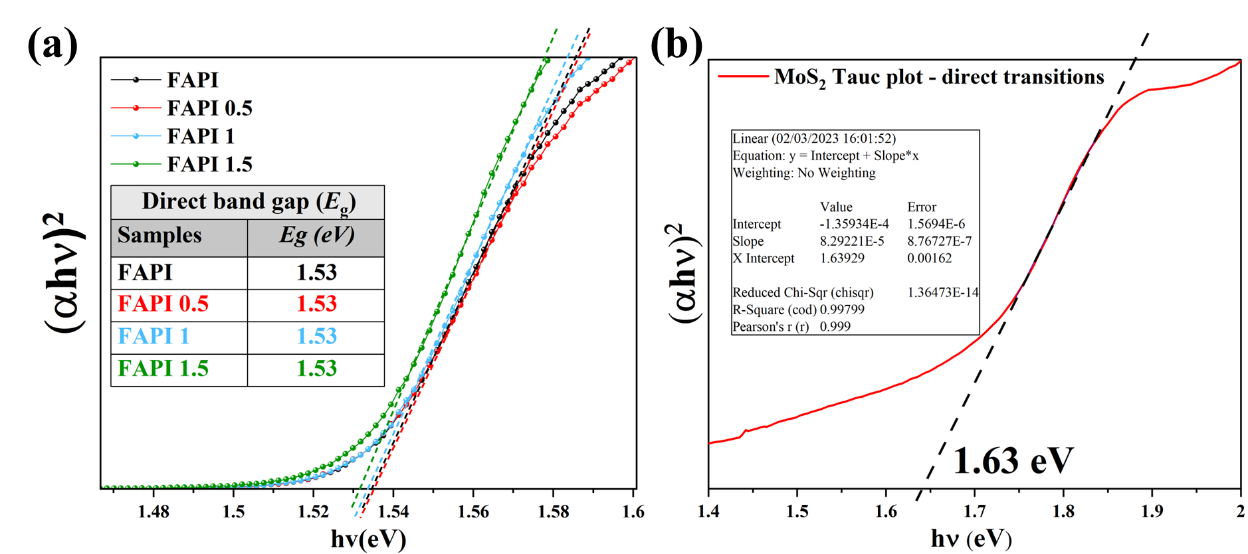


Figure S6: Direct transition Tauc plots for (a) FAPI and FAPI/MoS_2_ film and (b) MoS_2_ QDs.


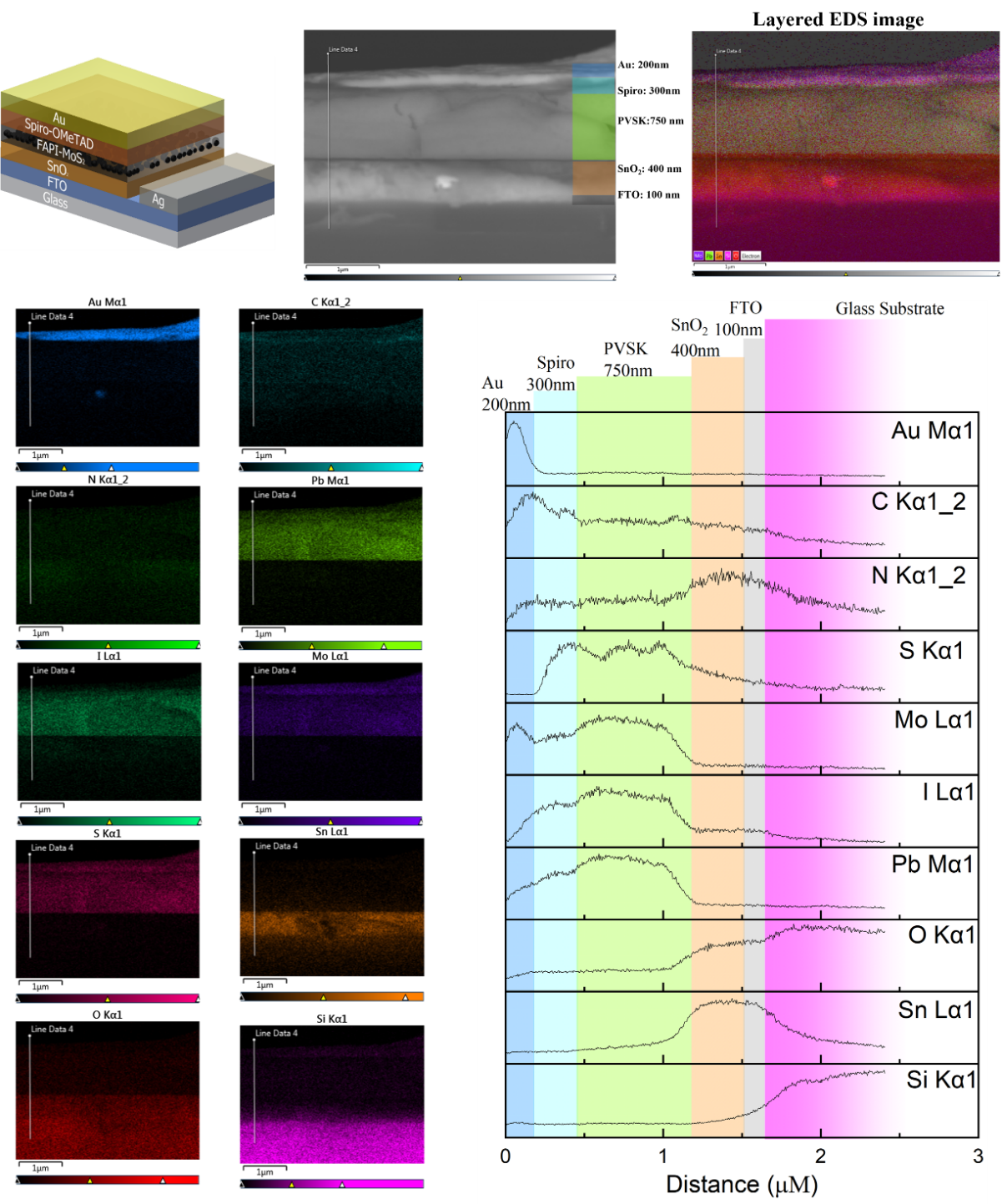


**(a)**

**(b)**

**(c)**

**(d)**

**(e)**

**(f)**

**(g)**

**(i)**

**(j)**

**(k)**

**(h)**

**(l)**

**(m)**

**(n)**


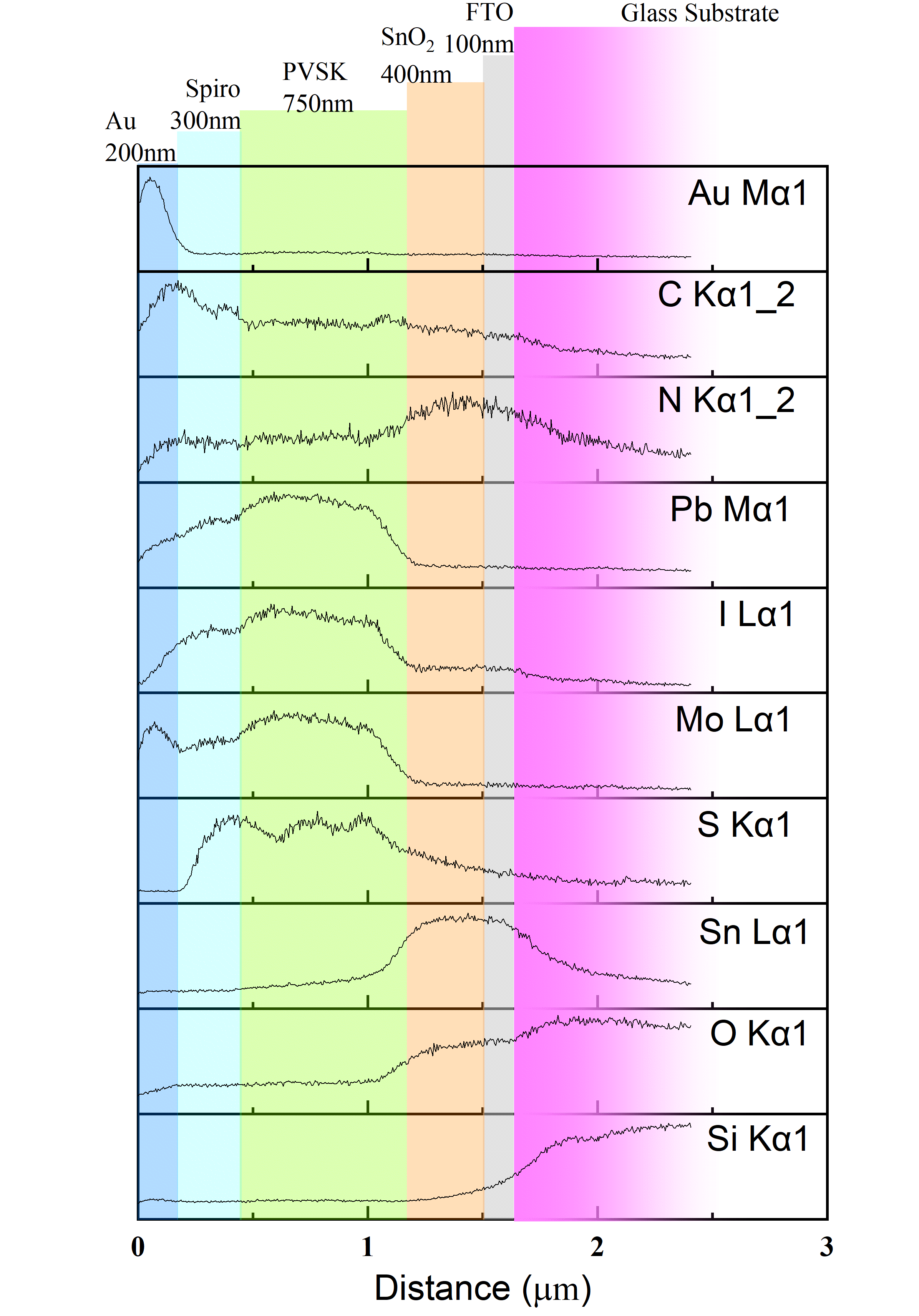


**(n)**

Figure S7: (a) Schematic diagram of the n-i-p type-I solar cell architecture with FAPI/MoS_2_ as active layer (PVSK). (b)SEM cross-section image of the solar cell and (c) shows the layered EDS colour maps highligthing solar cell layers. Individual EDS colour map of solar cell cross-section showing elements (d) gold, (e) carbon, (f) nitrogen, (g) lead, (h) iodine, (i) molybdeum, (j) sulfur, (k) tin, (l) oxygen and (m) silicon and (n) shows EDS line scan the stated individual elements.

Figure S8: (a) IQE-1 vs α-1 plot of FAPI and FAPI/MoS_2_ films for minority carrier diffusion length (L_diff_) calculations and (b) dark-current density measurements showing the comparison between FAPI and FAPI/MoS_2_ (FAPI 1) solar cells.


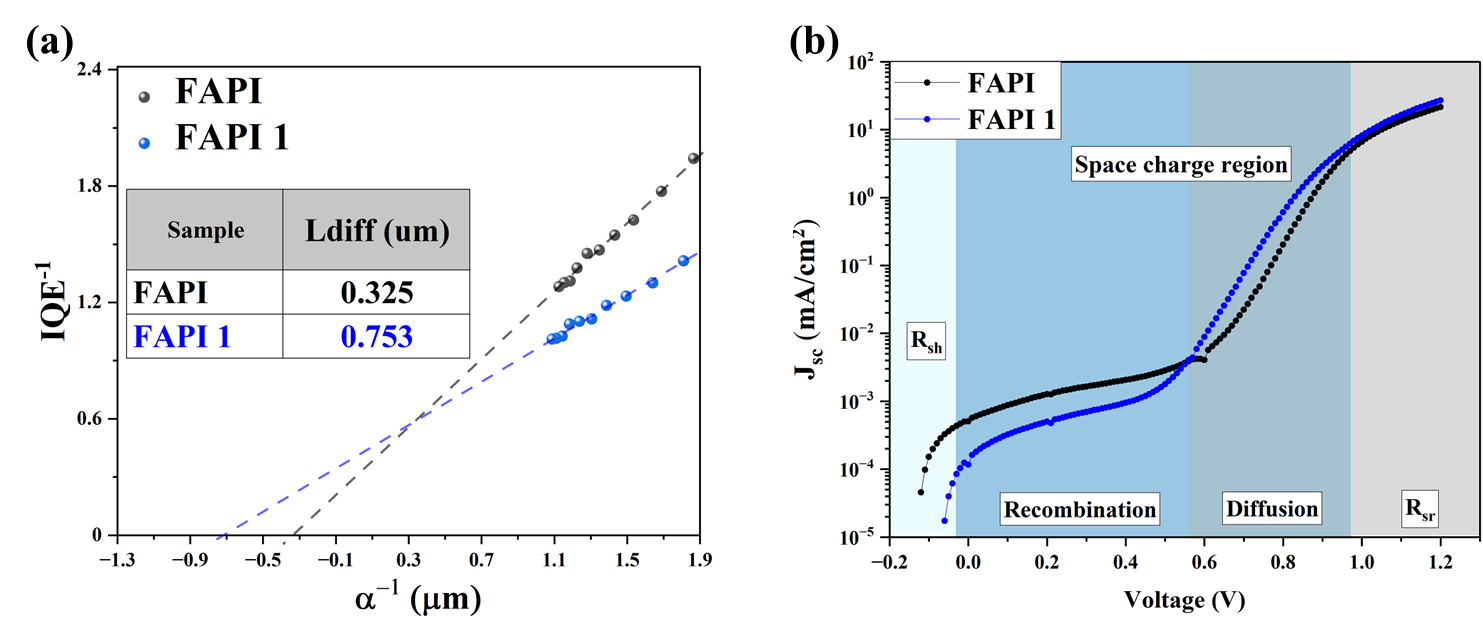

Supplement: Supplementary file 1 — Supplementary Information. [file 41598_2024_72037_MOESM1_ESM.docx]
